# Editorial: Unravelling the reality of COVID–19 cardiovascular complications: true myocarditis vs. myocardial injury—the role of a multilayered approach

**DOI:** 10.3389/fcvm.2024.1481667

**Published:** 2024-09-04

**Authors:** N. Wilmes, A. R. Vrettou, S. Lerakis, F. W. Asselbergs

**Affiliations:** ^1^ Department of Cardiology, CARIM School for Cardiovascular Diseases, Maastricht University Medical Centre (MUMC+), Maastricht, Netherlands; ^2^ 2nd Department of Cardiology, Attikon University Hospital, Haidari, Greece; ^3^ Mount Sinai Fuster Heart Hospital, Icahn School of Medicine at Mount Sinai, New York, NY, United States; ^4^ Department of Cardiology, Amsterdam Cardiovascular Sciences, Amsterdam University Medical Center, University of Amsterdam, Amsterdam, Netherlands; ^5^ Institute of Health Informatics, University College London, London, United Kingdom; ^6^ The National Institute for Health Research University College London Hospitals Biomedical Research Center, University College London, London, United Kingdom

**Keywords:** COVID—19, perimyocarditis, cardiovascular complications, post covid condition (PCC), vaccination, myocardial injury

**Editorial on the Research Topic**
Unravelling the reality of COVID–19 cardiovascular complications: true myocarditis vs. myocardial injury—the role of a multilayered approach

Since its emergence back in 2019, SARS-CoV-2 has infected almost 800 million people and has resulted in over 7 million fatalities (1). While SARS-CoV-2 primarily impacts the respiratory system, it is a multi-organ disease that manifests in various extrapulmonary ways, including the cardiovascular system (2). One of the ways it manifests is as myocardial injury, defined as a troponin level above the 99th percentile of the upper reference limit (3). The incidence of myocardial injury ranges broadly, and depends on the setting it is measured in. It ranged from 4.8% in a study including patients with only mild infection, to 54% in a study including critically ill participants (4). Patients with evidence of myocardial injury have a worse prognosis. This is due to a higher need for invasive ventilation and a higher in-hospital mortality (5, 6). The current research topic adds to this existing evidence, with a contribution from Yu et al. showing that elderly patients infected with the Omicron variant in 2022, still show evidence of myocardial injury. They also show that patients with myocardial injury are at a higher risk of requiring ICU admission. This shows that myocardial injury was not only a contributing factor during the early days of the pandemic, but still relevant with newer COVID-19 variants. Another contribution to this topic by Peiro et al. confirms the argument that myocardial injury irrespective of the underlying mechanism, is relevant throughout the different waves of the pandemic, by showing a prevalence of myocardial injury of 21% with a non-significant difference between the waves. Authors also showed an association between myocardial injury and preexisting cardiovascular risk factors. Finally, myocardial injury predicted 30-day all-cause mortality.

A non-frequent cardiovascular manifestation of COVID-19, is myocarditis in all its forms. Ranging from cardiac symptoms consistent with acute myocarditis, echocardiographic and positive cardiac magnetic resonance imaging (CMR) findings to fulminant myocarditis. One review estimates that 2.6% of COVID-19 cases are complicated by myocarditis (4). Another study, using a federated healthcare database, reports an incidence of 5% new-onset myocarditis (7). Details on the occurrence of myocarditis as a complication after COVID-19 are difficult to obtain, since many studies diagnosed myocarditis, based on ECG changes, an elevated serum troponin, echocardiographic changes or electrocardiographic changes. This diagnostic heterogeneity leads to doubts as to the commonality of myocarditis in COVID-19 patients (8). Endomyocardial biopsy (EMB) data are scarce, limited to case reports and a multicenter study of COVID-19 myocarditis that reported EMB data on 17 out of 112 patients diagnosed with acute COVID-19 myocarditis (9). The contribution by Liu et al. to this research topic looks at this discussion from a new perspective. Using mendelian randomization they did not find genetic evidence for a causal relationship between COVID-19 and a higher risk of peri-myocarditis.

Not only SARS-CoV-2 itself is related to myocarditis, but also mRNA vaccines were suspect. This happened after several individuals in Israel were reported to have myocarditis after receiving the Pfizer vaccine (10). One systematic review and meta-analysis shows that the relative risk for myocarditis was seven times higher in adults infected with COVID-19 than for those that were vaccinated (11). Pareek et al. adds to this research topic with their study into post-vaccination myocarditis by reporting a rate of less than 0.1% when comparing the three most used vaccines.

COVID-19 is not just responsible for short-term complications but can also cause long term complications. This is known as the so called “post-COVID-19 condition” (PCC), defined as the continuation or development of new symptoms 3 months after the initial SARS-CoV-2 infection, with these symptoms lasting for at least 2 months with no other explanation (12). The incidence of post-COVID-19 symptoms differs since most research was done prior to the Delphi consensus, meaning that the timeframes and required symptoms for a person to be classified as having PCC varied (13). However, Ballering et al. reported that when correcting for pre-COVID-19 symptoms, approximately 12.7% of patients had symptoms that could be attributed to previous COVID-19 disease including cardiac related symptoms, such as chest pain (14). These symptoms raise questions whether COVID-19 also causes long-term cardiac complications. Sasko et al. contribute to this question with their research by comparing hospitalizations in a region in Germany for arterial hypertension and myocardial infarction pre- and post-pandemic. They did not find a difference in rate of hospitalizations. Other papers, however, do find signs of COVID-19 related cardiac dysfunction during long-term follow-up. One study found cardiac dysfunction in 6.9% of patients at 8–16 weeks follow-up (15). Another study investigated patients for a median of 13 months after infection and found an incidence of 3% myocardial infarction. One last paper found a 2.8 times increased risk of ischemic heart disease over a 12 month follow-up in patients recovered from COVID-19 compared to those that were never infected (16).

Comparison is difficult due to the heterogeneous methodology of studies investigating post-COVID-19 outcomes. Some studies report CMR or echocardiographic data, while others report solely symptoms, and others concentrate on hard clinical endpoints. This makes it difficult to interpret the true burden of cardiovascular complications after COVID-19. There is a need for more studies investigating the long-term cardiovascular effects of COVID-19, preferably in a standardized, prospective manner. However, these studies need to define and monitor clinical endpoints, but also the cardiovascular aspects of PCC. Data collected in this way will be essential to our collective readiness for a future pandemic, which might not be far away. Currently, the avian flu is causing concern. It has spread from birds to dairy herds in the United States, forewarning a possible spread to humans (17, 18). At the same time, the World Health Organization just declared the spread of mpox on the African continent as a public health emergency of international concern (19).

This research topic containing the previously mentioned articles, takes us one step closer to understanding the relationship between COVID-19 and cardiovascular complications.
